# What is the Pisa Syndrome? A review

**DOI:** 10.1192/j.eurpsy.2022.1872

**Published:** 2022-09-01

**Authors:** H. Santos, E. Dornelles, J. Pereira, A. Vieira

**Affiliations:** 1 Centro Hospitalar Psiquiátrico de Lisboa, Psiquiatria Forense, Lisbon, Portugal; 2 Centro Hospitalar Lisboa Norte, Serviço De Psiquiatria, Lisbon, Portugal; 3 Centro Hospitalar Psiquiátrico de Lisboa, Clinica 6, Lisbon, Portugal; 4 Centro Hospitalar Psiquiátrico de Lisboa, Ccsmo, Lisbon, Portugal

**Keywords:** Pisa Syndrome, review

## Abstract

**Introduction:**

Pisa syndrome (PS) is a type of dystonia of rare occurrence, first described in 1972 as an adverse effect of neuroleptic agents. It is used to describe a postural abnormality that includes trunk flexion in the coronal plane and axial rotation, which improves in the supine position.

**Objectives:**

In this work, we aim to conduct a brief review of Pisa Syndrome aetiology, pathophysiology and treatment.

**Methods:**

A non-systematic search was conducted through the PubMed database for “pisa syndrome”. Articles were screened for relevant information on PS aetiology, pathophysiology and treatment.

**Results:**

Pisa syndrome has been associated as an adverse effect of multiple drugs from different classes, mainly antipsychotics, dopaminergic agents and cholinesterase inhibitors. The underlying mechanisms are not yet fully understood. Nevertheless, one of the most consensual hypothesis considers PS as a consequence of a cholinergic-dopaminergic imbalance that can be caused by antipsychotic treatment. Some factors have been associated with increased risk for developing PS such as old age and polypharmacy. PS appears to be better treated with the reduction or interruption of the agent(s) associated with its onset.

**Conclusions:**

Despite its low incidence, Pisa syndrome can occur as a side effect of a number of different medications and the identification of the trigger-drug is fundamental so it can be reduced or interrupted in order to treat this condition.

**Disclosure:**

No significant relationships.

